# Comparing newly developed SNP barcode panels with microsatellites to explore population genetics of malaria parasites in the Peruvian Amazon

**DOI:** 10.3389/fgene.2024.1488109

**Published:** 2024-12-19

**Authors:** Luis Cabrera-Sosa, Mahdi Safarpour, Johanna Helena Kattenberg, Roberson Ramirez, Joseph M. Vinetz, Anna Rosanas-Urgell, Dionicia Gamboa, Christopher Delgado-Ratto

**Affiliations:** ^1^ Laboratorio de Malaria: Parásitos y Vectores, Laboratorios de Investigación y Desarrollo, Facultad de Ciencias e Ingeniería, Universidad Peruana Cayetano Heredia, Lima, Peru; ^2^ Instituto de Medicina Tropical “Alexander von Humboldt”, Universidad Peruana Cayetano Heredia, Lima, Peru; ^3^ Malaria Research Group (MaRch), Global Health Institute (GHI), Family Medicine and Population Health Department (FAMPOP), Faculty of Medicine, University of Antwerp, Antwerp, Belgium; ^4^ Department of Biomedical Sciences, Institute of Tropical Medicine, Antwerp, Belgium; ^5^ Laboratorio ICEMR-Amazonia y Enfermedades Emergentes, Laboratorios de Investigación y Desarrollo, Facultad de Ciencias e Ingeniería, Universidad Peruana Cayetano Heredia, Lima, Peru; ^6^ Section of Infectious Diseases, Department of Internal Medicine, Yale School of Medicine, New Haven, CT, United States; ^7^ Departamento de Ciencias Celulares y Moleculares, Facultad de Ciencias e Ingeniería, Universidad Peruana Cayetano Heredia, Lima, Peru

**Keywords:** malaria molecular surveillance, microsatellites, SNP, NGS, population genetics, population structure, AmpliSeq

## Abstract

**Introduction:**

Malaria molecular surveillance (MMS) can provide insights into transmission dynamics, guiding national control programs. We previously designed AmpliSeq assays for MMS, which include different traits of interest (resistance markers and *pfhrp2/3* deletions), and SNP barcodes to provide population genetics estimates of *Plasmodium vivax* and *Plasmodium falciparum* parasites in the Peruvian Amazon. The present study compares the genetic resolution of the barcodes in the AmpliSeq assays with widely used microsatellite (MS) panels to investigate population genetics of Amazonian malaria parasites.

**Methods:**

We analyzed 51 *P. vivax* and 80 *P. falciparum* samples from three distinct areas in the Loreto region of the Peruvian Amazon: Nueva Jerusalén (NJ), Mazan (MZ), and Santa Emilia (SE). Population genetics estimates and costs were compared using the SNP barcodes (*P. vivax*: 40 SNPs and *P. falciparum:* 28 SNPs) and MS panels (*P. vivax*: 16 MS and *P. falciparum*: 7 MS).

**Results:**

The *P. vivax* genetic diversity (expected heterozygosity, *He*) trends were similar for both markers: *He*_MS_ = 0.68–0.78 (*p* > 0.05) and *He*_SNP_ = 0.36–0.38 (*p* > 0.05). *P. vivax* pairwise genetic differentiation (fixation index, F_ST_) was also comparable: F_ST-MS_ = 0.04–0.14 and F_ST-SNP_ = 0.03–0.12 (pairwise *p* > 0.05). In addition, *P. falciparum* genetic diversity trends (*He*_MS_ = 0–0.48, *p* < 0.05; *He*_SNP_ = 0–0.09, *p* < 0.05) and pairwise F_ST_ comparisons (F_ST-MS_ = 0.14–0.65, F_ST-SNP_ = 0.19–0.61, pairwise *p* > 0.05) were concordant between both panels. For *P. vivax*, no geographic clustering was observed with any panel, whereas for *P. falciparum*, similar population structure clustering was observed with both markers, assigning most parasites from NJ to a distinct subpopulation from MZ and SE. We found significant differences in detecting polyclonal infections: for *P. vivax*, MS identified a higher proportion of polyclonal infections than SNP (69% vs. 33%, *p* = 3.3 × 10^−5^), while for *P. falciparum*, SNP and MS detected similar rates (46% vs. 31%, *p* = 0.21). The AmpliSeq assay had a higher estimated per-sample cost compared to MS ($183 vs. $27–49).

**Discussion:**

The SNP barcodes in the AmpliSeq assays offered comparable results to MS for investigating population genetics in *P. vivax* and *P. falciparum* populations, despite some discrepancies in determining polyclonality. Given both panels have their respective advantages and limitations, the choice between both should be guided by research objectives, costs, and resource availability.

## 1 Introduction

In 2022, nearly 250 million malaria cases were estimated worldwide, mainly concentrated in low and middle-income countries of Africa, Latin America (LATAM) and Southeast Asia (World Health Organization, 2023). Many countries are making efforts to eliminate malaria in the next years by implementing National Malaria Control and Elimination Programs (NMCP/NMEPs).

The World Health Organization (WHO) states in the Global Strategy 2016–2030 that transforming malaria surveillance into a key intervention in NMCP/NMEPs is a crucial pillar for malaria elimination (World Health Organization, 2021). Surveillance can strengthen NMCP/NMEPs by providing information on transmission dynamics, drug resistance evolution, etc., that can be used to propose strategies for control and elimination and inform decision-making (Dalmat et al., 2019).

Malaria molecular surveillance (MMS) can support traditional epidemiologic surveillance based on microscopy or rapid diagnostic tests (RDT) to understand transmission dynamics (Golumbeanu et al., 2023). MMS also has the potential to be implemented into NMCP/NMEPs to contribute to the disease control efforts (Noviyanti et al., 2020). Scenarios where genetic surveillance data of *Plasmodium vivax* and *P. falciparum* parasites can inform malaria strategies have been identified (Dalmat et al., 2019). In that sense, population genetics can be used for these “use cases” to measure transmission intensity (Pava et al., 2020), identify parasite relatedness and gene flow (Brown et al., 2021; Schaffner et al., 2023), distinguish imported cases and transmission chains (Trimarsanto et al., 2022), characterize outbreaks and transmission foci (Wasakul et al., 2023) and determine genetic diversity and population structure (Nderu et al., 2019; Sy et al., 2022).

Microsatellites (MS) are short tandem repeat loci found in approximately 10% of the *Plasmodium* genome (Mathema et al., 2020). MS are high polymorphic and neutral markers (not under selection pressure), and have been used extensively to determine the population’s genetic changes (Anderson et al., 2000; Ferreira et al., 2007; Imwong et al., 2007). More recently, single nucleotide polymorphisms (SNPs) have gained popularity for population genetics analysis, not only for malaria but also for other infectious diseases and in conservation studies (Brashear and Cui, 2022; Theissinger et al., 2023). Although SNPs have low allelic diversity, they have high prevalence in the genome, allowing larger panels, have low level of homoplasy, are easy to be standardized with good reproducibility across different laboratories (Zimmerman et al., 2020). SNP genotyping can be performed as panels, also called SNP barcodes, and many platforms have been developed in the last decade for *P. vivax* (Baniecki et al., 2015; Kattenberg et al., 2022; Trimarsanto et al., 2022) and *P. falciparum* (Daniels et al., 2008; Campino et al., 2011; Harrison et al., 2024).

We have developed and validated two amplicon-based next-generation sequencing AmpliSeq assays (multiplexed amplicon sequencing technology that enables the targeted amplification and sequencing of multiple regions of the genome in a single assay) for malaria molecular surveillance in Peru (Pv AmpliSeq v2 Peru and Pf AmpliSeq v1 Peru assay) (Kattenberg et al., 2023a; Kattenberg et al., 2024). To serve for many uses cases, these assays include molecular resistance and other relevant markers (such as *pfhrp2/3* genes in *P. falciparum*) and Peru-specific SNP barcodes (composed of 28 SNPs for *P. falciparum*, and 41 for *P. vivax*) for population genetics analysis. These assays enabled to identify predominant *P. falciparum* lineages with low genetic diversity, double *pfhrp2/3* deletion (Kattenberg et al., 2023a), to determine a high heterogeneous *P. vivax* transmission in settings with different characteristics (Kattenberg et al., 2024), and to study the microepidemiology of an indigenous community with high prevalence and persistence of *P. vivax* malaria and a *P. falciparum* outbreak (Cabrera-Sosa et al., 2024).

As new MMS platforms like the AmpliSeq assays become available, it is relevant to assess whether SNP barcode resolution is comparable to that of the MS panels previously used (Fola et al., 2020; Ghansah et al., 2023). In this study, we aimed to compare the genetic resolution of SNP barcodes in the AmpliSeq assays and MS panels for assessing *P. vivax* and *P. falciparum* populations in the Peruvian Amazon. Since both the SNP and MS panels utilized neutral markers, this allowed for a direct comparison between the two methods. Specifically, we compared genetic diversity and differentiation metrics and population structure parameters between SNP/AmpliSeq and MS panels. We found both panels provided comparable results for assessing genetic diversity and population structure of *P. vivax* and *P. falciparum* populations in Peru, each with unique advantages. Also important, the comparable results between SNP barcodes and MS panels allow consistent comparison of population parameters across different studies with either panel. This capability supports the potential implementation of these panels into the Peru’s NMEP to characterize the temporal and spatial dynamics of malaria transmission and enhance monitoring and implement more effective intervention strategies for malaria elimination.

## 2 Materials and methods

### 2.1 Study sites and samples

The samples used in this study were previously collected from three geographically distinct locations within the Loreto region of the Peruvian Amazon (Supplementary Table S1). The first location, Nueva Jerusalen (NJ), is an indigenous community located northeast of Loreto, approximately 100 km from Iquitos, accessible primarily by river transport for 2 days. The *P. vivax* population in NJ showed modest genetic diversity, while *P. falciparum* had a lower diversity. There were also significant levels of genetic differentiation between parasites from NJ and other remote areas with some temporal clustering observed in the *P. falciparum* population, linked to an outbreak in February 2020 (Cabrera-Sosa et al., 2024). Samples from NJ were collected through active case detection (ACD) in November 2019 (*P. vivax*, n = 48; *P. falciparum*, n = 17) and passive case detection (PCD) from December 2019 to May 2020 (*P. vivax*, n = 20; *P. falciparum*, n = 49) (Cabrera-Sosa et al., 2024).

The second location, Mazan (MZ), is a riverine district situated around 50 km southeast of Iquitos. Due to its proximity to the urban center, MZ experiences higher human mobility, which may influence malaria transmission. Referring to the previous studies, the *P. vivax* population showed little differentiation between MZ and other communities, suggesting a high level of gene flow and connectivity (Manrique et al., 2019). In contrast, the *P. falciparum* population exhibited great genetic differentiation, indicating that transmission between different areas is limited (Kattenberg et al., 2023a). Samples from MZ were collected by two population-based cross-sectional surveys during 2018 (*P. vivax*, n = 13; *P. falciparum*, n = 10) (Villasis et al., 2021; Rosado et al., 2022).

Lastly, SE is a remote riverine community located 30 km south of Mazan, accessible only by boat. Its isolation makes it an important site for studying malaria in remote communities. In this community, the *P. vivax* population displayed moderate genetic diversity. In contrast, *P. falciparum* population showed slightly lower diversity with low to moderate levels of population differentiation (Manrique et al., 2015; Cabrera-Sosa et al., 2024). Samples were collected from SE by ACD and PCD in 2016 (*P. falciparum*, n = 14).

Blood samples (dried filter papers for NJ and SE, or packed red blood cells for MZ) were collected for molecular diagnosis. Also, thick and thin blood smears were taken for light microscopy diagnosis in the health post of each community. Regardless of the symptoms, treatment was provided for any positive microscopy result, following the national guidelines (Ministerio de Salud, 2015).

### 2.2 Ethics statement

All sample collections were approved by the Institutional Ethics Committee at Universidad Peruana Cayetano Heredia (UPCH) (SIDISI codes: 64024, 101518 and 102725). Written informed consent was obtained from all participants in MZ and SE. Samples from NJ were collected as part of the Ministry of Health (MINSA) activities and then transferred to our team for research purposes. This study was also approved by Institutional Ethics Committee at UPCH (SIDISI 207543). All methods were performed following the MINSA guidelines and regulations.

### 2.3 Sample processing

DNA was extracted using EZNA^®^ Blood DNA (Omega Bio-tek, United States), following the manufacturer’s protocol with an elution volume of 50 µL. Molecular diagnosis was performed by real-time PCR (qPCR). Samples from MZ were diagnosed using a SYBR Green-based protocol (Mangold et al., 2005). A TaqMan-based assay (Rougemont et al., 2004) was used for samples from NJ and SE.

### 2.4 AmpliSeq assays

Eighty-one *P. vivax* and 86 *P. falciparum* samples with parasitemia greater than 5 parasites/μL were genotyped with the Pv and Pf AmpliSeq Peru assays (Supplementary Figure S1). Library preparation was performed as previously described (Kattenberg et al., 2023a; Kattenberg et al., 2023b; Kattenberg et al., 2024). In brief, 7.5 µL of DNA was amplified with two primer pools. Then, digestion, indexes ligation, library amplification and cleaning steps were performed. Libraries were quantified using Qubit High sensitivity DNA kit (Invitrogen), pooled, diluted to 2 nM and denatured with NaOH to 7pM. Finally, the denatured library pool was loaded on a MiSeq system (Illumina) for 2 × 300 paired-end sequencing (Miseq Reagent Kit v3, Illumina) with 5% PhiX spike-in (Illumina).

FASTQ files were processed using an analysis algorithm as previously described (Kattenberg et al., 2023b). After trimming, reads were aligned to the reference genome (PvP01 v46 or Pf3D7 v44 from PlasmoDB, https://plasmodb.org/plasmo/app). Then, variants were called, generating individual gVCF files per each sample, which were combined to call genotypes jointly. After, variants were hard filtered and annotated. Finally, Variant Call Format (VCFs) containing only the Peru SNP barcode in each AmpliSeq assay (28 for *P. falciparum*, 41 for *P. vivax*) were created (Kattenberg et al., 2023a; Kattenberg et al., 2024).

The diploid genotype table (GT) was extracted from the SNP barcode VCF and manually reformatted into a haploid GenAlEx file (Peakall and Smouse, 2012). We constructed multi-locus genotypes (MLG) by combining the alleles across all loci from the barcode and assumed 2 MLG per sample. Then, when a sample had at least one heterozygous position, two MLG were created for GenAlEx file. The reference allele of each heterozygous position was kept in the first “dominant” MLG, and the alternative allele in the other “secondary” MLG, maintaining the homozygous positions in both.

### 2.5 MS genotyping

In total, 62 *P. vivax* and 90 *P. falciparum* samples were genotyped by using *Plasmodium* species-specific MS panels (Supplementary Figure S1). For *P. vivax*, a 16 MS panel (11.162, Ch2.121, 14.297, Ch2.152, Ch14.3021, Ch14.2986, Ch14.3010, Ch2.122, MS9, 13.239, Ch14.2981, MS6, MS4, MS15, 3.502, MS20), previously developed by our team, was used (Manrique et al., 2019). Samples were classified according to their parasitemia and followed a different processing before MS genotyping. Samples with >230 parasites/μL (par/μL) were diluted to 75–100 par/μL for the MS amplification. On the other hand, samples between 40 and 230 par/μL were amplified by selective whole genome amplification (sWGA) using the enzyme phi29 DNA Polymerase (NEB) before the genotyping. Finally, DNA from samples between 15 and 40 par/μL was reextracted, mixed with the first extraction and used for posterior PCRs (Manrique et al., 2019). Each MS was amplified by conventional PCR. In each reaction, one of the primers was labeled with a fluorophore for later identification. The master mix consisted of 1U of the AccuStart II Taq DNA polymerase enzyme (QuantaBio), 2.5 μL of PCR buffer, 1.5 mM MgCl_2_, 200–400 nM of each primer (depending on the panel) and 2.5 μL of DNA. The amplification protocol consisted of an initial denaturation at 95°C for 5 min, followed by 40 cycles of 30s at 94°C, 30s at 55°C–67°C (Tm depending on the MS) and 30s at 72°C. The last step was a final extension at 70°C for 15 min (Manrique et al., 2019).

In the case of *P. falciparum*, a panel of seven MS (TA1, Poly-α, PFPK2, TA109, 2490, C2M34, C3M69), previously applied to Peruvian samples (Akinyi et al., 2013; Bendezu et al., 2022) was used. Three amplification protocols were used, including two direct PCR (for TA109, Poly-α, PFPK2, C2M34 and C3M69) and one semi-nested PCR (for TA1 and 2490) (Nair et al., 2003; Roper et al., 2003). The master mix consisted of 1X Promega Master-mix (Promega), 400 nM of each primer and 2 µL of DNA or 1 µL of the product of the first PCR. For all cases, extracted DNA was used directly, without dilution or sWGA.

In both *P. vivax* and *P. falciparum*, positive controls and no-template controls were included in the reactions. After MS amplification, the PCR products were diluted and mixed with formamide and the GeneScan 500 LIZ marker (Applied Biosystems). Finally, the mixes were analyzed by capillary electrophoresis on an ABI Prism 3130 (Applied Biosystems). Chromatograms were analyzed with the Microsatellite Analysis software (Applied Biosystems). A quality control of the size marker was performed and the list of peaks per sample was downloaded.

With this data, a semi-automated analysis was performed to select true alleles. First, the peak with the highest relative fluorescence units (RFU) value (height) in the no-template controls was used as a threshold and all peaks with lower height in samples were discarded. After, the peak with the highest height was considered the main allele for each MS and sample. Secondary alleles were considered if their peak height was at least 1/3 of the main allele, and their size was not within ± 2bp of the main allele. As a final validation step, we manually compared the selected alleles in each sample to the positive control, discarding any allele when a discrepancy in the shape or pattern of peaks was detected. If a previously selected main allele was discarded in this process, the remained allele with the highest height was reclassified as the new main allele.

The final allele table was reformatted manually into a haploid GenAlEx file (Peakall and Smouse, 2012). For subset analysis, we separated the data of samples with secondary alleles into 2 MLG in the GenAlEx formatted file, with the main alleles kept in the first “dominant” MLG, and the secondary alleles in the other MLG, maintaining the rest of MS alleles in both.

### 2.6 Sample and markers quality control

We applied some inclusion criteria for the population genetics analyses (Supplementary Figure S1). First, to ensure comparability, we selected samples with genotyping data from both AmpliSeq and MS (*P. vivax*, n = 62; *P. falciparum*, n = 86). Then, samples with missing data in more than 25% of panels (SNP or MS) were excluded. 80/86 *P. falciparum* and 51/62 *P. vivax* samples passed this filter. Next, we kept markers with less than 25% missing data. Using this criteria, 2/28 positions from the *P. falciparum* SNP barcorde and 1/16 MS from the *P. vivax* panel were excluded. At the end, the final genotyped data included 80 *P. falciparum* samples with 7 MS (105 MLG) and 26 SNP (117 MLG), and 51 *P. vivax* samples with 15 MS (86 MLG) and 40 SNP (68 MLG) (Supplementary File S1).

### 2.7 Data and population genetic analysis

We assessed the inclusion of the secondary MLG in the analysis by comparing the genetic differentiation of MLG sets containing: Set 1) samples with only one MLG, Set 2) dominant MLG of all samples, Set 3) dominant MLG of all samples and secondary MLG of samples with in only one secondary allele, and Set 4) all dominant and secondary MLG of all samples. Fixation index (Fst) was calculated using hierfstat package in R (Goudet et al., 2022). Genetic differentiation (Fst) between all these sets were close to 0 (Supplementary Figure S2), indicating no genetic differences between them. Therefore, all MLG for all samples were included.

We calculated population genetic parameters using each SNP and MS panel for *P. vivax* and *P. falciparum* samples. Genetic diversity was measured as expected heterozygosity (He) and calculated using the formula: He = [n/(n − 1)][1 − Σp^2^], where n is the number of genotyped samples and p is the frequency of each allele at a given locus (Nei and Roychoudhury, 1974; Serrote et al., 2020). Genetic differentiation was expressed as pairwise Fst values and 95% confidence interval (95% CI), calculated based on Weir and Cockerham’s Method using the diveRsity package in R (Weir and Cockerham, 1984; Keenan et al., 2013). Population structure was explored using principal component analysis (PCA) (Jolliffe and Cadima, 2016) and the software STRUCTURE v2.3 (Pritchard et al., 2000). PCA was performed using the “prcomp” function in stats R-package. STRUCTURE determined the most likely number of clusters (K). Runs were performed exploring K from 1 to 10 (20 iterations each), assuming a mixture model, correlated allele frequencies, and using sampling location as priors (LOCPRIOR option). Each run had a burn-in period of 50,000 iterations followed by 150,000 Markov Chain Monte Carlo (MCMC) iterations. The most likely K was defined by calculating the rate of change of K (ΔK) (Evanno et al., 2005), using the R package pophelper (Francis, 2017). The complexity of infection (COI) was used to assess the proportion of polyclonal infections (multiple clones in one sample). Samples with 2 MLG (i.e., at least one heterozygous SNP genotype for the AmpliSeq data or at least one secondary allele in any MS) were considered polyclonal. For comparison, we also determined COI with the within-sample F (Fws) statistic using all biallelic SNPs detected by the AmpliSeq assays (*P. vivax*: 2923 SNPs, *P. falciparum*: 963 SNPs) using the moimix package in R. Samples were considered a monoclonal infection when the Fws was ≥ 0.95 (Auburn et al., 2012).

### 2.8 Statistical analysis

He values across different locations were compared performing the Kruskal-Wallis rank sum test. Dunn’s *post hoc* tests were used to perform pairwise comparisons between all possible pairs of groups to determine which specific groups are significantly different from each other. Comparison of He results obtained with MS and SNP were calculated using the correlation coefficient using Spearman’s rank correlation. Pairwise Fst from SNP and MS data were compared using Wilcoxon Signed-Rank Test. Proportions of mono/polyclonal infections according to SNP barcode, MS and Fws were compared for each species (*P. falciparum* and *P. vivax*) using the chi-squared test. Cohen’s Kappa coefficient was used to assess the agreement between SNP and MS in determining the complexity of infection. All statistical analyses were performed in R Studio (version 2022.12.0) using R version 4.2.2. *P*-values < 0.05 were considered significant. The Bonferroni correction was applied to adjust the *p*-values when performing multiple pairwise comparisons.

### 2.9 Cost estimates

Costs associated with AmpliSeq and MS genotyping for 96 samples (and controls) were estimated, including materials (reagents and kits) and time-personnel costs. All material prices were calculated based on quotations and invoices from Lima, Peru, from 2022. The cost does not consider DNA extraction, molecular diagnosis and plastic labware because those methods are similar for both procedures. For *P. vivax* MS genotyping, we assumed that 60% of samples required sWGA (Manrique et al., 2019). Total costs included portion of materials used exactly for 96 samples. Separately, we calculated the cost of sequencing (MiSeq) and capillary electrophoresis (ABI Prism 3130), including equipment and annual maintenance.

Personnel cost per day (60 USD/working day) was calculated considering monthly salary of 1,200 USD and 20 working days in one month. Number of working days in each activity was based on practical experience. Costs in Peruvian Nuevos Soles (PEN) were converted into United States Dollars (USD) using the exchange rate of 1 PEN = 0.26 USD.

## 3 Results

### 3.1 AmpliSeq SNP barcode and MS genotyping performance

For *P. vivax*, only the reference allele was detected for the SNP PvP01_13_v1_32509. The median minor allele frequency (MAF) for the rest of 40 SNPs was 0.27 [range: 0.02–0.46, mean ± standard deviation (sd): 0.25 ± 0.14]. All *P. vivax* samples had ≤25% missing genotypes in the barcode. In the case of *P. falciparum*, all alternative alleles at 28 SNPs were genotyped successfully. The median MAF was 0.19 (range: 0–0.48, mean ± sd: 0.20 ± 0.15). Two SNPs (Pf3D7_02_v3_694307 and Pf3D7_09_v3_231065) had >25% of missing data (57% and 66%, respectively) and were excluded for downstream analyses. A low proportion of samples (4.65%, 4/86) had >25% of missingness in the SNP barcode and were subsequently excluded.

The amplification efficiency of both MS panels is shown in Supplementary Figure S3. For *P. vivax*, MS had low proportion of missing data (3.2%–22.6%), except for Ch14.3010 (43.6%), which was excluded for later analyses. 52 (83.4%) samples had ≤25% missing genotypes. On the other hand, amplification efficiency in the *P. falciparum* MS panel was ≥90% (% missing data: 0%–10%). Only 5 samples (5.6%) with >25% of missingness were excluded.

### 3.2 Genetic diversity

**FIGURE 1:**
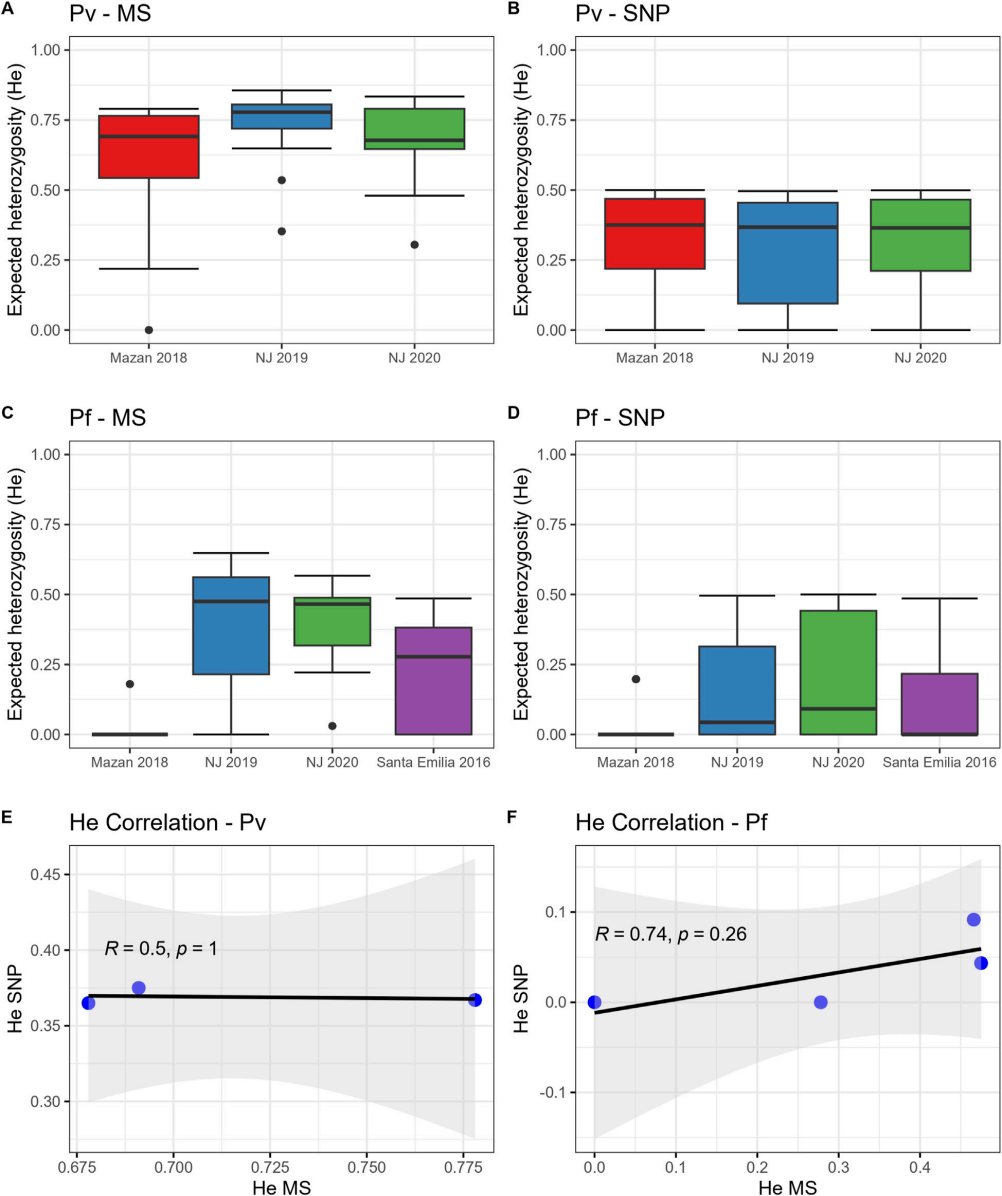
Expected heterozygosity (He) in *P. vivax* and *P. falciparum* populations using SNP and MS. He was calculated for *P. vivax* **(A, B)** and *P. falciparum* **(C, D)** using MS **(A, C)** or SNP **(B, D)**. The horizontal line within the boxes represents the median He from each panel (MS) or position (SNP) for all samples in each group. Dots outside the boxes represent the outlier data. In addition, Spearman’s correlations of He values between MS and the SNP barcode for **(E)** *P. vivax* and **(F)** *P. falciparum* populations were determined.

We found similar trends of population diversity (expressed as He) with both panels (MS or SNP) for both species (Figure 1; Supplementary Figure S4). *P. vivax* populations in MZ and NJ had similar genetic diversity when using MS (Figure 1A, median He: 0.68–0.73, *p* = 0.23) or SNP (Figure 1B, median He: 0.37–0.38, *p* = 0.80). We also noted that 2/40 (5%) of the SNP positions were fixed in all population (Supplementary Table S2).

On the other hand, *P. falciparum* isolates from MZ (median He: 0 with both markers) had lower diversity than parasites from NJ using MS (Figure 1C, median He: 0.36–0.37, *p* = 0.03–0.04) or SNP (Figure 1D, median He: 0.04–0.09, *p* = 0–0.03). Also, He values in *P. falciparum* samples from MZ and NJ were similar to SE isolates, determined by MS (median He: 0.28, *p* = 0.78–1) and by SNP (median He: 0, *p* = 0.36–1). Finally, around 40% of SNP positions (10/26) were fixed in these populations (Supplementary Table S2).

Despite the similar trends in the genetic diversity, we found no significant correlation between He values obtained by MS and SNP in the *P. vivax* (Figure 1E, R = 0.5, *p* = 1) and *P. falciparum* (Figure 1F, R = 0.74, *p* = 0.26) populations, although the correlation lacked statistical power as the degrees of freedom were low (1 and 2, respectively).

### 3.3 Genetic differentiation

**FIGURE 2:**
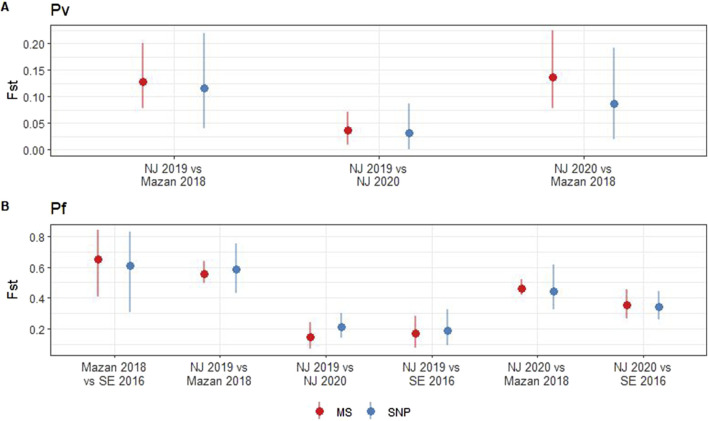
Pairwise fixation index (Fst) in *P. vivax* and *P. falciparum* populations using SNP and MS. Fst was calculated for *P. vivax* **(A)** and *P. falciparum* **(B)** using MS (red) or SNP barcode (blue). Pairwise Fst values (points) and 95% confidence intervals (lines) were estimated with 1000 bootstraps using the diveRsity package in R.

We observed no significant differences between the pairwise Fst values obtained using MS and SNP across the *P. vivax* (*p* = 0.34–0.85) and *P. falciparum* (*p* = 0.24–0.83) populations (Figure 2). In case of *P. vivax*, low differentiation of parasites within NJ was noted when using MS (Fst = 0.04, 95% CI: 0.01–0.07) or SNP (Fst = 0.03, 95% CI: 0–0.09) (Figure 2A). Moderate levels of differentiation were observed between MZ and NJ with MS (Fst = 0.13–0.14, 95% CI: 0.08–0.22) and SNP (Fst = 0.09–0.12, 95% CI: 0.02–0.22) (Figure 2A). In the *P. falciparum* population, the lowest Fst values were found between the two groups in NJ when using MS (Fst = 0.14, 95% CI: 0.07–0.24) and SNP (Fst = 0.19, 95% CI: 0.09–0.32). On the other hand, the highest Fst values was observed between MZ and SE isolates (Fst = 0.65, 95% CI: 0.41–0.84 with MS, Fst = 0.61, 95% CI: 0.31–0.83 with SNP) (Figure 2B). The consistency between the two markers suggests that both MS and SNP markers are capable of reliably capturing genetic differentiation in the Peruvian setting.

### 3.4 Population structure

**FIGURE 3:**
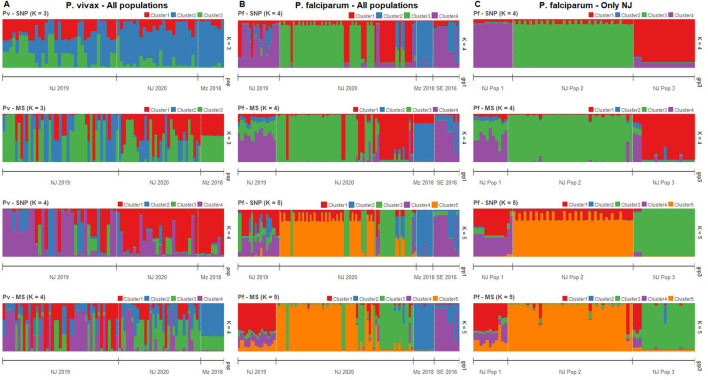
Clustering analysis by STRUCTURE in *P. vivax* and *P. falciparum* populations using SNP and MS. The graph depicts the clustering models when the isolates were assigned into different number of clusters (K). Each isolate is represented by a single vertical line broken into K colored segments, with lengths proportional to each of the K inferred clusters. **(A)** Clustering of *P. vivax* populations sorted by location at K = 3 and 4 when using SNP and MS. **(B)** Clustering of *P. falciparum* populations sorted by location at K = 4 and 5 when using SNP and MS. **(C)** Clustering of *P. falciparum* populations in NJ by previous classification according to (Cabrera-Sosa et al., 2024) at K = 4 and 5 when using SNP and MS.

Clustering patterns obtained from STRUCTURE were strikingly similar between MS and SNP in both species (Figure 3), although the most likely number of clusters (K) differed between panels. For *P. vivax*, the predicted best K were 2, 4, and 6 according to the SNP barcode, whereas the best K were 3 and 6 using MS (Supplementary Figure S5). For *P. falciparum*, the predicted best K using SNP were 3, 5 and 7, meanwhile the most likely K with MS were 2, 4, and 7 (Supplementary Figure S6). For comparison between MS and SNP, we plotted the assignation to 3, 4, and 6 clusters for *P. vivax* isolates (Figure 3A; Supplementary Figure S5) and to 4, 5 and 7 clusters for *P. falciparum* parasites (Figure 3B; Supplementary Figure S6).

For *P. vivax* populations, high admixture was observed in NJ, with all clusters present in both years. In contrast, all samples from MZ belonged to predominantly one cluster according to SNPs, while MS did not attribute to any single cluster but showed the same admixture pattern (Figure 3A; Supplementary Figure S5).

In the case of *P. falciparum*, strong clustering observed in all population, except in NJ 2019, which presented high admixture. Predominant clusters in NJ 2020 and MZ were different, and SE shared some clusters with NJ 2019 and MZ. SNPs also detected some admixed samples in NJ 2020, which were predicted to belong to a single cluster by MS (Figure 3B; Supplementary Figure S5).

Previously, we identified three genetic clusters (Pop 1 to Pop 3) in the *P. falciparum* population in NJ using all biallelic SNPs detected in the AmpliSeq assay based on results from PCA and supported by other analyzes such discriminant analysis of principal components (DAPC), identity by descent (IBD) network and phylogenetics (Cabrera-Sosa et al., 2024). Using STRUCTURE analysis, all those genetic clusters (Pop 1 to Pop 3) had distinct patterns with only the 28-SNP barcode or MS (Figure 3C; Supplementary Figure S6). In Pop 1, similar admixture clustering was frequent, except when K = 4 with SNPs were plotted. Different clusters were predominant in each Pop 2 and Pop 3 (Figure 3C; Supplementary Figure S6).

**FIGURE 4:**
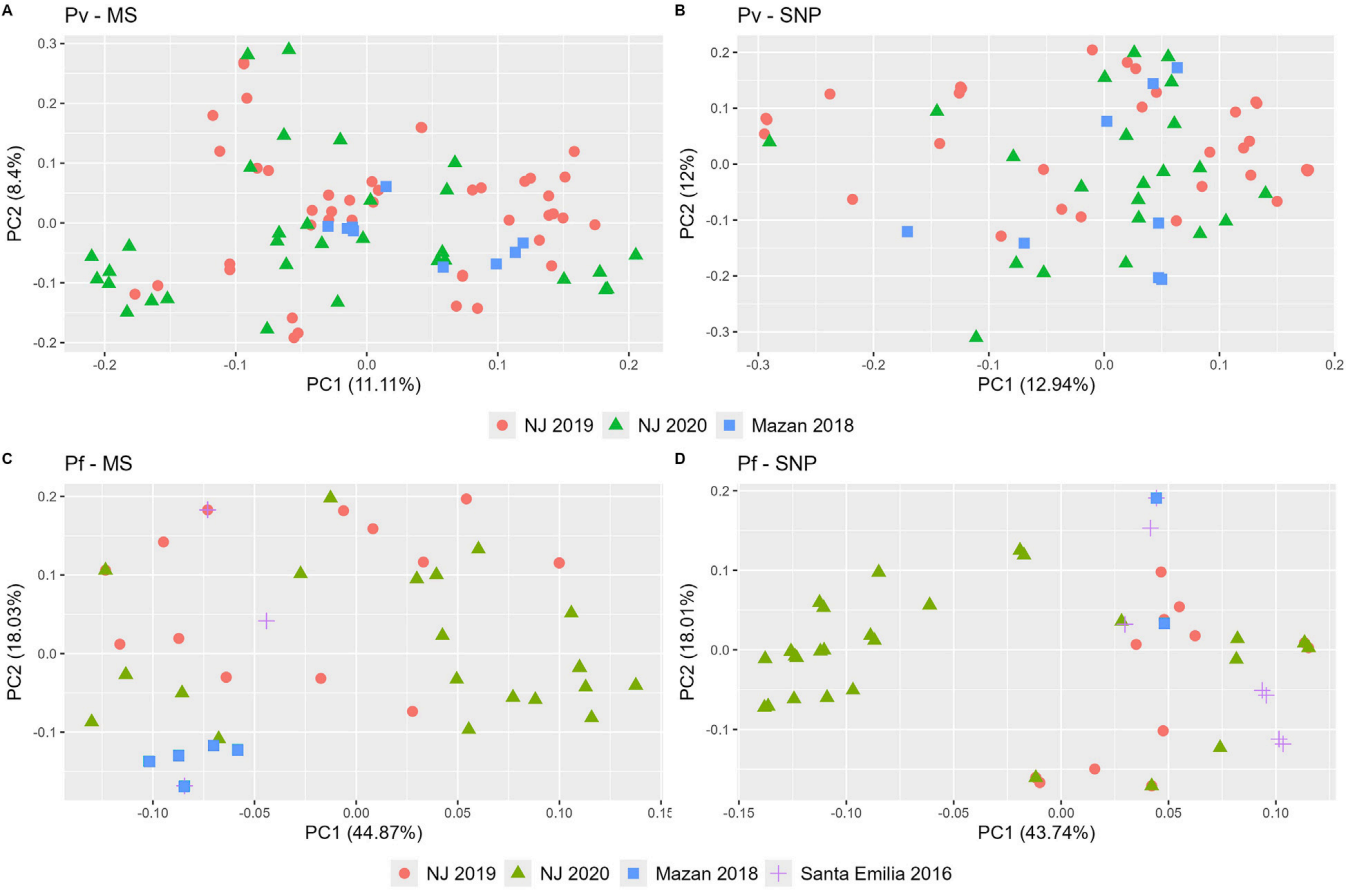
Principal component analysis (PCA) of *P. vivax* and *P. falciparum* populations using SNP and MS. PCA was performed for *P. vivax* **(A, B)** and *P. falciparum* **(C, D)** using MS **(A, C)** or SNP **(B, D)**. Each community is represented by a unique shape and color. Consistent shapes are used to identify the same community across both *P. vivax* and *P. falciparum* populations.

PCA analysis confirmed patterns observed in the STRUCTURE analysis, and overall similar clustering in both MS and SNP (Figure 4). For *P. vivax*, no clear geographical or temporal clustering was observed in either panel (Figures 4A, B). On the other hand, a stronger clustering of *P. falciparum* isolates of NJ 2020 was observed in SNP than with MS (Figures 4C, D). The variance explained by the first two principal components were similar for *P. vivax* (20% and 25% for MS and SNP, respectively) and *P. falciparum* (63% for MS, 62% for SNP).

### 3.5 Complexity of infection (COI)

We found differences in the proportion of polyclonal infections determined by the SNP barcode, MS and the Fws statistics (Table 1). For *P. vivax*, this proportion was higher with MS (69%) than with SNP and Fws (33% and 28%, respectively; *p* = 3.3 × 10^−5^). Five MS (MS9, MS15, Ch2.121, Ch2.122, Ch2.152) accounted for high proportion of samples with secondary alleles (26%–43%). For *P. falciparum*, the SNP barcode detected similar polyclonal infections as MS (46.2% and 31.2%, respectively, *p* = 0.21), but higher than Fws (29%, *p* = 0.043). Agreement between SNP barcode and MS was higher in *P. falciparum* (κ = 0.54, 95% CI: 0.36–0.72) than in *P. vivax* (κ = 0.23, 95% CI: 0.04–0.43).

**TABLE 1:** Proportion of polyclonal infections by SNP barcode, MS, and Fws in *P. vivax* and *P. falciparum* samples.

Population	*P. vivax* (n = 51)				*P. falciparum* (n = 80)			
	SNP barcode - AmpliSeq	MS	Fws	*p*-value	SNP barcode - AmpliSeq	MS	Fws	*p*-value
NJ 2019	8 (29.6%)	18 (66.7%)	6 (22.2%)	**3.3 × 10** ^**−5**^	8 (57.1%)	4 (26.8%)	3 (20.1%)	**0.01**
NJ 2020	7 (38.9%)	14 (77.8%)	6 (33.3%)		27 (60%)	20 (44.4%)	19 (42.2%)	
MZ 2018	2 (22.2%)	3 (33.3%)	2 (22.2%)		0 (0%)	1 (11.1%)	0 (0%)	
SE 2016					2 (16.7%)	0 (0%)	1 (8.4%)	
Total	**17 (33.3%)**	**35 (68.6%)**	**14 (27.5%)**		**37 (46.2%)**	**25 (31.2%)**	**23 (28.8%)**	

Samples were considered polyclonal with at least one heterozygous position (SNP barcode), secondary allele (MS) or Fws < 0.95. Bold values indicate the total number and proportion of polyclonal infections for each method and species. The p-value corresponds to the comparison among these total values for each species.

### 3.6 Cost estimations

We estimated higher costs associated with AmpliSeq compared to MS genotyping (Table 2). The estimated cost of the AmpliSeq was 183 USD/per sample, whereas using the MS panels was 49 USD/sample for *P. vivax* (16 MS) and 27 USD/sample for *P. falciparum* (7 MS). Notably, a large difference was observed for the personnel costs, with the AmpliSeq been 2 to 4 times cheaper than MS genotyping (5 vs. 11–19 USD/sample), as it was much faster (AmpliSeq: 6 working days, MS: 18 to 30 working days) (Supplementary File S2). Total cost for equipment and annual maintenance was higher with the MiSeq than ABI 3130 (190k vs. 160k USD), although when referring as external service, cost for AmpliSeq and *P. vivax* MS genotyping was similar (33 vs. 30 USD/sample) (Supplementary File S2).

**TABLE 2:** Estimated costs of AmpliSeq and MS genotyping for 96 samples.

	*P. vivax* or *P. falciparum* AmpliSeq			*P. vivax* MS (16 MS)			*P. falciparum* MS (7 MS)		
	Total cost (PEN)	Total Cost (USD)	Cost/sample (USD)	Total cost (PEN)	Total Cost (USD)	Cost/sample (USD)	Total cost (PEN)	Total Cost (USD)	Cost/sample (USD)
Materials	65,695	17,081	118	11,253	2,926	30	5,800	1,508	16
Personnel	1,846	480	5	6,923	1,800	19	4,154	1,080	11
Total	67,541	17,561	**183**	18,176	4,726	**49**	9,954	2,588	**27**

Bold values indicate the total cost per sample (in USD) for each genotyping method.

## 4 Discussion

The use of genomic tools for malaria parasite genomic surveillance has increased over the last decade. In the past, genetic panels such as MS were mainly used to characterize genetic population of malaria parasites, particularly using panels composed of relatively few MS, typically <20 (Sutton, 2013; Manrique et al., 2019; Gwarinda et al., 2021). More recently, the technological advancements in DNA sequencing enable researchers to analyze many loci at once. Due to their higher prevalence in the genome, potential to target functional regions, and the fact that MS regions often perform poorly in targeted sequencing (Rovira-Vallbona et al., 2023b), SNPs have been replacing MS in population genetic studies (Zimmerman et al., 2020; Neafsey et al., 2021). As new technologies involving SNP barcodes for surveillance appear, a comparison with MS genotyping could offer valuable insights into the similarity of output results and the limitations and advantages of each method, and their applications under different scenarios. In this study, we demonstrated good agreement between SNP barcodes from our novel AmpliSeq assays (Kattenberg et al., 2023a; Kattenberg et al., 2024) and MS panels for population genetic analyses in the same group of *P. vivax* and *P. falciparum* samples from the Peruvian Amazon, showing that similar conclusions on the transmission dynamics can be concluded with both panels.

In the case of the diversity metrics, both panels yielded relatively consistent trends. For *P. vivax*, He values were consistent across different study settings as shown in MS and SNP results. For *P. falciparum*, both panels agreed on the diversity differences between MZ and NJ. This observation shows that the SNP barcode and MS could capture a high portion of the genome-wide variation similarly, which is consistent with previous report in *Plasmodium* (Kattenberg et al., 2024) and other species (Zimmerman et al., 2020; Perez-Gonzalez et al., 2023). As expected, He values derived from MS were higher across all populations for both *P. vivax* and *P. falciparum* populations compared to SNP, explained by the higher theoretical maximum heterozygosity in each panel (He = 1 for MS vs. He = 0.5 for SNP) (Zimmerman et al., 2020). In both panels, *P. vivax* population was more diverse than *P. falciparum* in the Peruvian Amazon, and association of genetic diversity with transmission intensity (NJ > MZ) were observed in *P. falciparum*, but not in *P. vivax*, all consistent with reports in other locations (Barry et al., 2015; Auburn et al., 2021). In that sense, our results are crucial because genetic diversity can be reliably estimated with SNP or MS and the similar trends allow to compare data from different studies that have used those panels. Nevertheless, it is important to acknowledge that one limitation of our study is the low statistical power, primarily due to the small number of data points (e.g., correlation of He). Consequently, broader sampling across diverse populations is needed to determine if similar results can be consistently reproduced, thereby validating our findings.

Generally speaking, genetic differentiation estimates (Fst) from MS panels and the SNP barcode were similar in the *P. vivax* and *P. falciparum* populations. Except for the *P. vivax* isolates within NJ, most of the pairwise comparisons showed moderate or great differentiation with both MS and SNP. This consistency between the both panels for the genetic differentiation in *Plasmodium* was observed in other species (Zimmerman et al., 2020; Perez-Gonzalez et al., 2023), showing that the level of differentiation can be reliable determined using either MS panel or SNP barcode. This measure provides valuable insights into the population structure, helps estimate gene flow among parasite populations, and can identify potential parasite migration route between locations, which are correlated to human mobility in most of the cases (Escalante et al., 2015; Escalante and Pacheco, 2019; Tessema et al., 2019), although other factors such as migration rates and population sizes may also contribute to the genetic differentiation (Meirmans and Hedrick, 2011; Kitada et al., 2021). Unfortunately, other connectivity and relatedness analysis, such as identity by descent (IBD), can only be performed with the SNP barcode or the MS panels with at least 200 positions (genomic loci) (Taylor et al., 2019). However, by performing IBD with all biallelic SNPs detected in the AmpliSeq assays (n = 2,925), we have previously described the relatedness of malaria parasites in the Peruvian Amazon (Cabrera-Sosa et al., 2024; Kattenberg et al., 2024), showing the capability of these assays to perform further analysis, including IBD, when a larger number of SNPs are included.

The population structure analyses demonstrated that although similar clustering patterns were obtained, the SNP barcode provided greater precision, resulting more distinct grouped individuals than the more loosely clustered individuals observed with MS panels, particularly for *P. falciparum*. In that sense, we found different optimal number of clusters when using MS and SNP in *P. vivax* and *P. falciparum*, which is in concordance to other reports in mammals, reptiles, and amphibians (Camacho-Sanchez et al., 2020; Biello et al., 2022; Perez-Gonzalez et al., 2023). This variation can be explained by number of samples per population and number of loci, which strongly affect the K determination and population assignment. Also, as SNPs have fewer possible variations, a change in one of their alleles might have a bigger impact on the results, making it more efficient to detect clusters than with a change of an allele in the larger MS pool (Puckett and Eggert, 2016; Camacho-Sanchez et al., 2020). Regardless, both MS and SNP barcode could detect and differentiate the reported genetic clusters in the *P. falciparum* population in NJ (Cabrera-Sosa et al., 2024), making both panels suitable for identifying outbreaks. Once again, these findings suggest that both panels can be effectively used to investigate population structure.

The contrasting results between SNP barcode, MS, and Fws highlight the difficulties in COI determination (Zhong et al., 2018). For *P. vivax*, the proportion of polyclonal infections was higher with the MS panel than the SNP barcode, while for *P. falciparum*, the proportions were similar between both panels, but SNP barcode results were higher than calculations with Fws. Previous reports have shown that SNP barcodes may underestimate COI compared to MS in *P. falciparum* populations in Vietnam and Peru (Kattenberg et al., 2023a; Rovira-Vallbona et al., 2023a), in contrast to a correlation between MS and Fws outputs for COI in a *P. falciparum* population from Guinea (Murray et al., 2016). MS could detect additional genotypes than SNP as their higher mutation rate leads to more alleles and more information content per locus (Sutton, 2013; Puckett and Eggert, 2016). Although higher with MS, proportion of polyclonality determined by SNP barcode in the *P. vivax* population was similar to previous reports in the Peruvian Amazon (Delgado-Ratto et al., 2016; Manrique et al., 2019; Kattenberg et al., 2024). This discrepancy can be explained by the mutation rate and allelic richness associated with MS, which allows MS to detect additional genotypes per locus. In contrast, SNP barcodes, while highly informative, often have lower variability per locus, which can lead to an underestimation of COI. Additionally, *P. vivax* and *P. falciparum* often show different transmission dynamics, which could impact COI estimates. In that regard, several studies have demonstrated the correlation between COI and prevalence or transmission level in *P. vivax* and *P. falciparum* populations around the world (Escalante et al., 2015; Fola et al., 2017; Kattenberg et al., 2020; Lopez and Koepfli, 2021), with some exceptions mainly observed in *P. vivax* (Escalante and Pacheco, 2019; Auburn et al., 2021). In our study, the *P. vivax* transmission levels in NJ (high) and MZ (low) correlated with their proportion of polyclonal infections. This association was not observed with genetic diversity, showing that COI may be used as a more effective proxy for transmission intensity. As the SNP barcodes were not specifically designed for measuring COI, MS panel’s allelic richness, particularly of MS9, MS15, Ch2.121, Ch2.122 and Ch2.152, make them more suitable for COI determination’s studies in *P. vivax* populations (Sutton, 2013). Finally, other markers (such as *ama1*, *csp* and *cpmp* genes) can be used to determine COI in *Plasmodium* populations (Zhong et al., 2018; Wamae et al., 2022). As the *ama1* gene is targeted in our AmpliSeq assays, this marker may complement the SNP barcode to assess COI (Kattenberg et al., 2023a; Kattenberg et al., 2024).

Previous studies have compared SNP and MS panels in *Plasmodium* populations, reporting varied findings on which method offers better resolution. For example, SNPs have been shown to offer higher resolution than MS in *P. vivax* isolates from Papua New Guinea in 2012–14 (Fola et al., 2020). In contrast, SNPs and MS effectively clustered *P. falciparum* from Africa, but only SNPs correctly classified strains from South and Central America (Kanai et al., 2022). However, in another study, SNPs had lower genetic resolution than MS for identifying changes following an intervention in a *P. falciparum* population in Ghana (Ghansah et al., 2023). Finally, similar levels of genetic differentiation and population structured were observed with both SNP and MS in *P. falciparum* populations from Western Kenya (Lo et al., 2018). In our study, comparable results were obtained with SNPs and MS in *P. falciparum* and *P. vivax* populations in Peru, with slightly higher resolution using the first in some analysis, which allowed to compare evolution of population genetics parameters through space and time more precisely. The differences in the geographic scale may be one explanation for the differences to the previous reports. Here, we worked at a small geographic scale, primarly characterized as a setting nearing malaria elimination (Auburn et al., 2021). Another explanation is the design of the SNP barcodes, which was based on whole genomic available data (Kattenberg et al., 2023a; Kattenberg et al., 2024). As also previously reported, we showed some fixed SNP positions, particularly in the *P. falciparum* population, that may affect how much diversity the barcodes can capture. Now, with higher available up to date genomes from the region, the resolution of the SNP barcode could be increased. Other factors can include number of panels and sample size, different mutation rates, diverse transmission level, and changes in the informativeness over time and place, making it necessary to validate the panels in new scenarios (Delgado-Ratto et al., 2016; Manrique et al., 2019; Taylor et al., 2019).

Our findings highlighted that comparing the efficiency of SNP barcodes in the AmpliSeq assays and MS panels for malaria parasite population genetics is complex and varies depending on the type of analysis and the specific parasite species. Even if SNP and MS panels remained comparable in different scenarios and have similar limits of detection (15 and 6 par/μL) without difference between both species (Manrique et al., 2019; Kattenberg et al., 2023a), each panel has challenges for their implementation into NMCP/NMEPs that should be considered (Ruybal-Pesantez et al., 2024). MS are highly polymorphic and have low cost. However, their genotyping is difficult to be reproduced and be standardized between different laboratories, and involves long time for laboratory procedure and data analysis, making them less suitable for larger sample sets (Puckett and Eggert, 2016; Zimmerman et al., 2020). Also, results may be affected by subjectivity during allele determination, highlighting the need for automated methods to avoid these issues (Manrique et al., 2015; Barbian et al., 2018; Salado et al., 2021). On the other hand, key benefits of the AmpliSeq assays are their suitability for high-throughput and automated analysis, facilitating the rapid generation of big amount of genetic information. While notably more expensive than MS, these assays can also include more than 10 drug resistance associated genes and *pfhrp2/3* genes (Kattenberg et al., 2023a; Cabrera-Sosa et al., 2024; Kattenberg et al., 2024). In our study, the number of SNP markers was relatively modest (40 SNPs for *P. vivax* and 28 SNPs for *P. falciparum*) compared to other studies (Amambua-Ngwa et al., 2019; Phelan et al., 2023), which often use larger panels of SNPs. Nevertheless, our assay still provided a high level of genetic resolution for molecular surveillance of malaria at a smaller geographical scale, particularly within-country and regionally targeted areas of the Peruvian Amazon. In addition, the SNP barcode can be updated if necessary, allowing the inclusion of additional SNPs. Particularly, we will work on revising the barcode in the *P. falciparum* AmpliSeq assay, as now there are more available Peruvian and LATAM genomes. Also, the AmpliSeq assays become cheaper as the number of processed samples increases (Supplementary File S2). Currently, we are testing other reagents with the same panel to reduce the library preparation costs. However, they require specialized technology and bioinformatics expertise, which are not always particularly accessible in remote areas of low and middle-income countries. This requires a better distribution of sequencing infrastructure and priority efforts from the health authorities, as seen with other pathogens in Peru (Padilla-Rojas et al., 2021; Puyen et al., 2024).

In conclusion, cost-effective and scalable platforms should be incorporated into NMCP/NMEPs for molecular surveillance. Here, we showed that the SNP barcode in the Pv and Pf AmpliSeq assays provide similar results to classic MS genotyping in population genetics analyses such as patterns of transmission intensity and population structure. As both types of panels are comparable, the selection of the best option should be based on the research questions and practical limitations, including cost, available infrastructure, and feasibility (Kanai et al., 2022). In any case, while tackling the associated challenges, the implementation of novel NGS tools, such the AmpliSeq assays, into the surveillance system in Peru may be encouraged.

## Data Availability

The datasets presented in this study can be found in online repositories. The names of the repository/repositories and accession number(s) can be found in the article/Supplementary Material.
